# Screening for High-Risk Human Papillomavirus Reveals HPV52 and HPV58 among Pediatric and Adult Patient Saliva Samples

**DOI:** 10.3390/dj12030056

**Published:** 2024-02-29

**Authors:** Hunter Hinton, Lorena Herrera, Sofia Valenzuela, Katherine M. Howard, Karl Kingsley

**Affiliations:** 1 Department of Advanced Education in Orthodontics, School of Dental Medicine, University of Nevada-Las Vegas, 1700 W. Charleston Boulevard, Las Vegas, NV 89106, USA; hintoh2@unlv.nevada.edu; 2 Department of Clinical Sciences, School of Dental Medicine, University of Nevada-Las Vegas, 1700 W. Charleston Boulevard, Las Vegas, NV 89106, USA; 3 Department of Biomedical Sciences, School of Dental Medicine, University of Nevada-Las Vegas, 1001 Shadow Lane Boulevard, Las Vegas, NV 89106, USA

**Keywords:** clinical sampling, saliva screening, high-risk human papillomavirus (HPV), oral cancer

## Abstract

Previous research has demonstrated that the human papillomavirus (HPV) can infect a wide range of human tissues, including those within the oral cavity. High-risk oral HPV strains have been associated with the development and progression of oral cancers, including oral squamous cell carcinomas. Although many studies have examined the prevalence of the high-risk strains HPV16 and HPV18, far fewer have assessed the prevalence of other high-risk HPV strains. An approved study protocol was used to identify HPV52 and HPV58 among clinical samples (*n* = 87) from a saliva biorepository. Quantitative polymerase chain reaction (qPCR) and validated primers for HPV52 and HPV58 were used to facilitate this screening. This screening demonstrated that a total of *n* = 4/45 or 8.9% of adult saliva samples harbored high-risk HPV52, and *n* = 2/45 or 4.4% tested positive for high-risk HPV58. In addition, a total of *n* = 6/42 or 14.3% of the pediatric saliva samples tested positive for high-risk HPV, including *n* = 5/42 or 11.9% with HPV52 and *n* = 3/42 or 7.1% for HPV58. These data demonstrate the presence of the high-risk oncogenic HPV52 and HPV58 strains among both adult and pediatric clinical patient samples. More detailed longitudinal research must be conducted to determine whether this prevalence may be increasing or decreasing over time. In addition, these data strongly support public health prevention efforts, such as knowledge and awareness of the nine-valent HPV vaccine covering additional high-risk strains, including HPV52 and HPV58.

## 1. Introduction

Many studies have demonstrated that the human papillomavirus (HPV) may be capable of infecting a wide range of human tissues, although it has been mostly demonstrated as the causative oncologic agent in cervical cancers and other related dysplasias [1,2]. Recent studies have demonstrated that HPV may be capable of infecting other types of epithelial tissues, including those in the breast and lung [3,4]. However, the most commonly detected sites for HPV infection other than cervical cancers may be found in the oral cavity and oropharynx—more specifically among oral dysplasias and oral squamous cell carcinomas [5,6].

Most of the focus of previous investigations has involved specific oncogenic high-risk strains of HPV, which are associated with the development and progression of many types of cancers, such as cervical cancers [7,8]. The most prevalent high-risk oncogenic strains from these sites that have been identified are HPV16 and HPV18, which have been closely associated with the majority of cervical as well as oral cancer cases [9,10,11]. Although many previous studies have examined the prevalence of high-risk strains HPV16 and HPV18, far fewer have evaluated the prevalence of the other high-risk oncogenic HPV strains, including HPV52 and HPV58—which are among the HPV strains now included in the modified nine-valent HPV vaccine [12,13,14].

The original quadrivalent (Gardasil) HPV vaccine covering the low-risk HPV6 and HPV11 strains as well as the high-risk HPV16 and HPV18 strains was modified in 2016 to the new nine-valent HPV vaccine (9vHPV) that still covers the most common low-risk and high-risk HPV strains implicated in the overwhelming majority of documented HPV-related diseases, as well as additional high-risk HPV strains [15,16]. The HPV strains comprising the modified vaccine continue to include the low-risk HPV 6 and 11 strains, responsible for more than 90% of anogenital warts, and provide protection against these sexually transmitted infections [17,18]. However, in addition to the HPV 16 and HPV18 strains responsible for the majority of cervical and oral cancers, additional cancer-causing high-risk HPV strains identified from epidemiologic studies of cervical and other epithelial tissues, such as HPV31, HPV33, HPV45, HPV52, and HPV58, are now included [19,20,21].

In fact, new evidence has suggested that HPV52 and 58 may be of particular concern to oral health professionals and oral epidemiologists [22,23,24]. Although most of the research into these high-risk strains has focused on cervical infections, there is evidence that these strains may be involved in the oncogenesis and transformation of oral infections and may mediate or modulate oral carcinogenesis [25,26,27]. Although previous studies at this institution have explored the high-risk oral prevalence of HPV strains 16 and 18 among children and adults, only one study to date has assessed additional high-risk strains, such as HPV31, 33, and 35, and no study to date has evaluated the presence of HPV52 and HPV58 among this patient population [28,29]. 

In addition, due to the non-invasive nature of saliva as a diagnostic fluid, many clinicians and public health researchers have begun to review existing biorepositories of previously collected samples to enable more thorough and comprehensive evaluations of oral health conditions, including oral microbial prevalence [30]. These salivary samples may be capable not only of providing important information regarding the composition of oral microbial constituents, including viruses, bacteria, and fungi, but they may also be an important tool to evaluate and assess other metabolic and diagnostic biomarkers for overall health, such as levels of glucose, inflammatory cytokines, and markers of oncogenesis and transformation that may provide the potential for monitoring these conditions without invasive blood sampling [31]. Recent reviews have demonstrated that saliva as a diagnostic medium may be effective in the detection of low-level and nascent oral infections, such as those presented during the SARS-CoV-2 pandemic, which has greatly strengthened the evidence base regarding the utilization of these samples for further analysis into the oral health of patient populations [32].

On the basis of the paucity of information regarding the oral epidemiology of these strains and their potential importance in understanding the role of high-risk HPV infection, the overall goal of this study was to evaluate the prevalence of HPV52 and 58 within a biorepository of existing clinical saliva samples originally taken from the pediatric and adult patient clinic populations. 

## 2. Materials and Methods

### 2.1. Protocol Approval

The investigation was approved by the Institutional Review Board and the Office for the Protection of Research Subjects (OPRS) at the University of Nevada, Las Vegas (UNLV) under protocol 1619329-1. The samples used in this study were previously collected by investigators under the publication, “The Prevalence of Oral Microbes in Saliva from the University of Nevada, Las Vegas School of Dental Medicine (SDM) Pediatric Adult Clinical Population”. The inclusion criteria for this study consisted of patients from ages 5 to 75. Pediatric assent from the patient with informed consent from the parent or guardian was required for any children under the age of 18. In adults 18 years or older, informed consent was obtained. The exclusion criteria were defined as any patients who refused the pediatric assent or informed consent and any samples from individuals who were not patients of the UNLV School of Dental Medicine. 

### 2.2. Original Sample Collection Protocol

The original convenience sample collection for the investigation involved all volunteer participants from whom pediatric assent and/or informed consent were obtained. Patients were then provided with a sample collection tube and were asked to provide 5.0 mL of unstimulated saliva. A random, non-duplicated number was created for each collection tube, ensuring that there was no identifying information specific to the patient. A demographic log of each generated number was made to identify the patient’s age, race or ethnicity, sex, and orthodontic status. The saliva samples were transferred and stored at −80 °C in a biomedical repository. 

### 2.3. Sample Processing and Analysis

From the salivary biorepository, a total of *N* = 87 met the inclusion criteria for this retrospective analysis. To isolate the DNA, phenol:chloroform extraction was used. To do this, the samples were first thawed and then vortexed. Using a sterile microcentrifuge tube, 500 µL of each saliva sample was combined with 500 µL of TRIzol DNA isolation reagent and 200 µL of chloroform from Invitrogen (Waltham, MA, USA) prior to incubation on ice for 15 min. The centrifugation of samples was performed at 12,000× RCF at a temperature of 4 °C for 15 min using the refrigerated microcentrifuge (Model 5425) from Eppendorf (Hamburg, Germany). 

To precipitate the DNA, approximately 400 µL of the upper aqueous layer was transferred to a sterile microcentrifuge tube, combined with isopropanol from Invitrogen (Waltham, MA, USA), and centrifuged. The isopropanol was then removed, leaving a DNA-containing pellet, which was washed with ethanol from Invitrogen (Waltham, MA, USA) and centrifuged for another 10 min. Finally, after removing the ethanol, the DNA was resuspended with 100 µL of nuclease-free water obtained from Fisher Scientific (Waltham, MA, USA). Using the NanoDrop 2000 spectrophotometer from Fisher Scientific (Waltham, MA, USA) at absorbances of A260 and A280 nm, both the DNA quantity and quality of each sample were analyzed. Samples with a final DNA quantity of >10 ng/µL and a quality of A260:A280 ratio of >1.65 were deemed sufficient and were then screened using qPCR. 

### 2.4. Quantitative Polymerase Chain Reaction (qPCR) Screening

The samples that met the above criteria for DNA quantity and quality were screened using qPCR for the high-risk HPV strains 52 and 58. In each reaction, there was 2.0 µL of sample DNA, 5.0 µL of nuclease-free water, 1.5 µL of forward primer, 1.5 µL of reverse primer, and 15 µL of Fast SYBR Green Master Mix from Applied Biosystems (Waltham, MA, USA) using the following primers:

Positive control

Glyceraldehyde 3-phosphate dehydrogenase (GAPDH)

GAPDH forward: 5′-ATCTTCCAGGAGCGAGATCC-3′

GAPDH reverse: 5′-ACCACTGACACGTTGGCAGT-3′

Screening primers

HPV52 forward: 5′-AAAGCAAAAATTGGTGGACGA-3′

HPV52 reverse: 5′-TGCCAGCAATTAGCGCATT-3′

HPV58 forward: 5′-GGCATGTGGATTTAAACAAAAGGT-3′

HPV58 reverse: 5′-TCTCATGGCGTTGTTACAGGTTAC-3′

### 2.5. Statistical Analysis

An appropriate step for any retrospective study is the calculation of the minimum sample size necessary, which can be estimated using the sample-limiting step of the DNA extraction recovery rate from the saliva samples. Using the manufacturer’s estimate of 90–95% efficiency, the expected maximum experimental difference of 10% or 0.10 was determined for this process. This information was subsequently used to calculate the minimum sample size using a power (p) of 0.90 combined with a significance level of alpha = 0.95 to estimate a minimum sample size of *N* = 50 saliva samples needed for inclusion in the current study. 

Analysis of any differences in the percentages between categorical variables, such as ethnicity, race, and sex, between the overall clinic population and the studied samples were analyzed with Chi-square. The qPCR screening results were analyzed and displayed as simple statistics such as percentages and are shown in the accompanying tables. The samples that tested positive or negative for HPV were analyzed using Fisher’s exact test, which may be the most appropriate analysis for the calculation and estimation of p-values for results with any individual category that has a sample size smaller than *N* = 50; GraphPad Prism software version 8 (San Diego, CA, USA) was used. For the parametric data comparisons, such as patient age, two-tailed Student’s *t*-tests were performed, with statistical significance determined using an alpha level of 0.05. 

## 3. Results

A total of *N* = 87 usable samples were obtained from both pediatric and adult patients within the saliva biorepository. The demographic characteristics from the adult patient samples (*n* = 45) were analyzed, revealing that slightly less than half were males (43.7%), which closely matched their overall distribution in the main patient clinic population (49.2%) and was not significantly different, *p* = 0.3172 (Table 1). Furthermore, the proportion of minority patient samples was greater than two-thirds (68.9%), which did not differ significantly from the proportion of these patients in the overall patient clinic (65.4%), *p* = 0.4017. Finally, the average age of the samples included in the study was found to be 41.2 years, which closely matched the overall age within the clinical population of 42.3 years, *p* = 0.711. 

**Figure 1:**
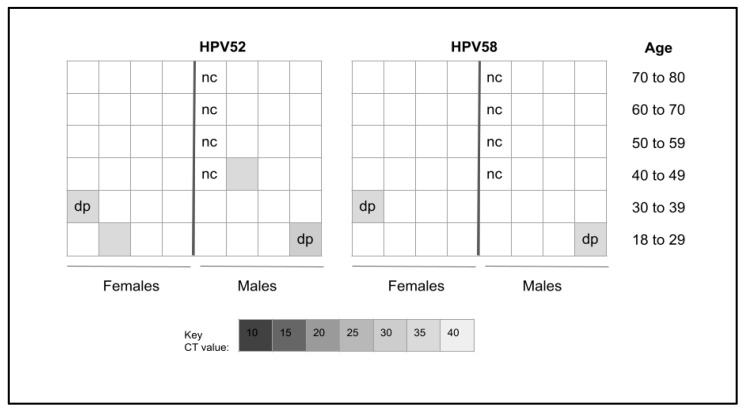
Heatmap analysis of adult sample qPCR screening. Four samples or 8.9% (*n* = 4/45) tested positive for HPV52 or HPV58, which were evenly distributed among males and females. Both samples testing positive for HPV58 were also positive for HPV52. Three of the four HPV-positive samples were within the recommended vaccination or catch-up range (9–26 or 27 to 45 years). nc = negative control, dp = double positive.

The adult samples identified from the salivary biorepository were subsequently evaluated for the presence of HPV52 or 58 (Figure 1). These data revealed that *n* = 4/45 or 8.9% of these samples demonstrated the presence of HPV52, HPV58, or both. More specifically, an equal number of samples derived from females (*n* = 2) and males (*n* = 2) harbored HPV52. In addition, only two samples (one female, one male) also harbored HPV58, which were among the HPV52-positive samples. Three of the four HPV-positive adult samples were within the CDC recommendation vaccination age (9–26 years) or catch-up range (27–45 years) for the nine-valent HPV vaccine.

A detailed analysis of the adult patient study sample demographics revealed that the HPV-positive samples were equally divided between males (50%) and females (50%), which approximated the distribution of males (42.9%) and females (56.1%) among the HPV-negative samples, *p* = 0.8418 (Table 2). In addition, the percentage of HPV-positive samples from minority (75%) and non-minority (25%) patients was also similar to the distribution of HPV-negative samples from minority (70.7%) and non-minority (29.3%) patients, *p* = 0.8573. Finally, the proportion of HPV-positive samples from patients within the vaccination or catch-up range (9 to 26 and 27 to 45) was greater (75%) than among the HPV-negative samples (53.4%) but was not statistically significant due to the small sample size, *p* = 0.7869.

Pediatric samples were also selected from the salivary biorepository, *n* = 42 (Table 3). The demographic analysis of the samples derived from pediatric patients (*n* = 42) revealed that fewer than half came from male patients (42.9%), which did not differ from the overall distribution of males within the pediatric patient clinic population (47.2%), *p* = 0.4229. The analysis of demographics regarding ethnic or racial sample data demonstrated that the samples from non-White or minority patients represented more than three-fourths (83.3%), which was higher than the proportion of these patients within the pediatric patient clinic population (75.3%), *p* = 0.0647. Finally, the average age of the samples included in this study was found to be 12.3 years, which was higher than the average age in the overall pediatric clinical population of 10.4 years, *p* = 0.2331. 

**Figure 2:**
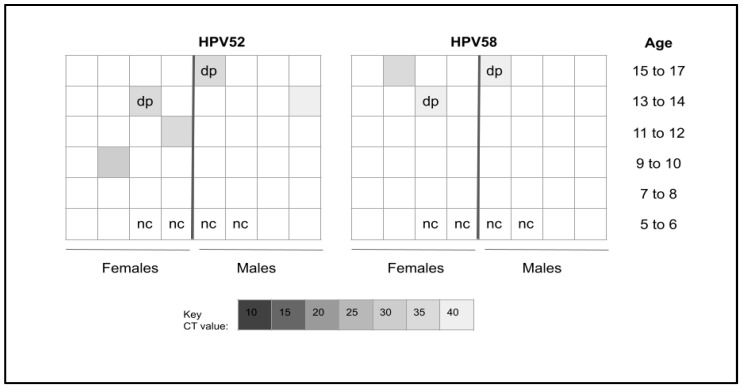
Heatmap analysis of pediatric study sample qPCR screening. Six samples or 14.3% (*n* = 6/42) tested positive for HPV52 or HPV58, with two samples harboring both HPV52 and HPV58. More females (*n* = 3) than males (*n* = 2) were found with HPV52 and also with HPV58 (females, *n* = 2; males, *n* = 1). All of the HPV-positive samples were within the recommended vaccination or catch-up range (9–26 years). nc = negative control, dp = double positive.

Pediatric patient samples from the salivary biorepository were then screened for the presence of HPV52 or 58 (Figure 2). These data revealed that *n* = 6/42 or 14.3% of these samples harbored HPV52, HPV58, or both. More specifically, more samples derived from females (*n* = 3) than males (*n* = 2) harbored HPV52. Similarly, more samples derived from females (*n* = 2) than males (*n* = 1) harbored HPV58. In addition, two samples (one female and one male) harbored both HPV52 and HPV58. All of the HPV-positive pediatric samples were within the CDC recommendation vaccination age (9–26 years) for the nine-valent HPV vaccine.

More detailed demographic analysis of the study samples from pediatric patients revealed that the HPV-positive samples were not equally divided between males (33.3%) and females (66.7%), but the distribution was not significantly different from the distribution of males (44.4%) and females (55.6%) among the HPV-negative samples due to the small sample size, *p* = 0.6852 (Table 4). Furthermore, the small proportion of HPV-positive samples from minority (66.7%) and non-minority (33.3%) patients was also not significantly different from the distribution of HPV-negative samples from minority (80.6%) and non-minority (19.4%) patients, *p* = 0.5928. Finally, although the proportion of samples with HPV from patients within the vaccination or catch-up range (9 to 26 years) was greater (100%) than that among the HPV-negative samples (66.7%), this was not statistically significant, *p* = 0.1589.

## 4. Discussion

The main study objective was to assess the prevalence of high-risk oncogenic strains HPV52 and 58 among adult and pediatric clinical saliva samples from an existing saliva biorepository. These data clearly demonstrated that both HPV52 and HPV58 were present among adults, which confirms a previous report of oral HPV52 among adults in other patient populations and represents one of the first studies to evaluate the oral prevalence of HPV58 within this patient population [33]. In addition, this study screened pediatric samples, revealing the presence of oral HPV52 and HPV58 among this patient population; it is only the second study to evaluate non-HPV16 and non-HPV18 oral strains among this pool of pediatric clinic patients [32].

These data are critically important as oral health professionals and epidemiologists strive to determine the extent of oral HPV infections among the general population. In particular, many studies have assessed oral HPV infection and risk among pediatric patients and have primarily focused on high-risk strains HPV16 and HPV18 covered by the original quadrivalent vaccine [34,35]. In previous studies from this group evaluating pediatric oral HPV, it was observed that HPV16 and HPV18 were found in approximately 9.2% of patient samples [29,30]. However, a more recent longitudinal analysis of additional samples collected over the past decade found that the high-risk oral HPV prevalence was actually increasing year over year, from an initial baseline of 5.7% in 2010 to 18.1% in 2020. As only one other study has screened for non-HPV16 and non-HPV18 strains in this pediatric patient population (covering HPV31, HPV33, and HPV35) and found that 21.9% harbored one or more of these strains, the data from this current study regarding HPV52 and HPV58 and their prevalence of 14.3% reveals even more patients who might benefit from the new nine-valent HPV vaccine, which includes these additional high-risk HPV strains [36,37].

This information is also important to oral healthcare and public health professionals, as recent evidence has demonstrated that vaccine hesitancy (particularly related to HPV among pediatric patients) has been increasing in recent years despite strong evidence for the efficacy and safety of both the quadrivalent and nine-valent HPV vaccines [38,39,40]. In fact, other recent evidence has also documented the increase in vaccine hesitancy and skepticism about vaccine safety and efficacy over recent years among children, adolescents, and their parents [41,42]. One of the most effective and important methods for addressing vaccine hesitancy may be current information regarding HPV prevalence and infections among the patient population, such as that presented in this study, which demonstrates the relevance and importance of vaccination against high-risk oral HPV strains even in very young patients [43,44,45].

Although these findings are significant, there are some intrinsic limitations found among retrospective studies that should also be considered. For example, this was an analysis of existing samples obtained from one-time sampling (cross-sectional collection); it does not provide any longitudinal or temporal information regarding the incidence of these high-risk strains over time and does not indicate whether these current findings represent an increase or decrease in these oral HPV strains [28,29]. In addition, as a retrospective study of existing biorepository samples, limited demographic data regarding these patients were available (sex, age, race, or ethnicity), and no other health or behavioral data were collected with the original saliva samples, such as HPV vaccination status or engagement with smoking or vaping products [46,47,48]. Moreover, the limited number of samples available from the biorepository for this retrospective study may be an additional factor that should be considered. Moreover, this patient population consists of primarily low-income and minority patients using federal assistance programs, such as Medicaid, and it may represent different challenges related to health awareness, the availability of vaccination programs, and other barriers to accessing care that must also be considered [49,50].

However, the significance of these results to clinical practice cannot be overstated. Although most oral HPV infections are cleared between 18 and 24 months, clearance rates involving infection with high-risk oncogenic HPV strains, such as HPV52 and HPV58, may actually be much lower [51,52]. In addition, recent evidence has also shown that HPV clearance may be reduced by other comorbidities and treatments, including antipsychotic and antidepressant medications, which have been increasingly common prescriptions among Western industrialized countries [53,54,55]. Furthermore, although prevalence studies are lacking, two new studies from Hong Kong and the French West Indies have found that HPV52 may be the most commonly detected oral high-risk HPV strain (even among the control, non-cancer patient population), with oral prevalence ranging between 1.5% and 33% [56,57].

Despite the lack of evidence regarding the incidence or prevalence of oral dysplasia and cancer among the pediatric population, a recent systematic review of pooled data from 60 studies found that of the 146 identified cases of pediatric HPV-related oral lesions and 153 cases of pediatric oral cancer, no record or report of HPV vaccination was found [35]. This information is particularly important when combined with new evidence that demonstrates that HPV vaccination may be effective in reducing HPV infection and HPV-related lesions specifically in the oral cavity [58,59,60]. Although there may be many other contributing factors that predispose patients to the acquisition and development of HPV-related oral infections, such as diet and oral hygiene, the most important factor identified to prevent high-risk HPV infection and disease is HPV vaccination [61,62,63].

## 5. Conclusions

Due to the paucity of information regarding HPV52 and HPV58 prevalence among any patient population, this study provides significant evidence that these high-risk strains are present among both pediatric and adult populations [32,33]. In addition, these data also demonstrated that the majority of samples with HPV were derived from patients within the recommended HPV vaccination range, providing significant information for health professionals and public health advocates on the urgent need to disseminate current knowledge about the benefits of the nine-valent HPV vaccine and the potential risks of avoidance for this low-cost and effective prevention method [36,37,38,39,40].

## Figures and Tables

**Table 1:** Analysis of adult patient study sample demographics.

Demographic of Adult Patient Samples	Study Samples	UNLV-SDM Clinic	Statistical Analysis
Sex (Adult)			
Adult Males	44.4% (*n* = 20/45)	49.1% (Adult clinic)	X^2^ = 1.000, d.f. = 1
Adult Females	55.6% (*n* = 25/45)	50.9% (Adult clinic)	*p* = 0.3172
Ethnicity/Race (Adult)			
White/Caucasian	31.1% (*n* = 14/45)	34.6% (Adult clinic)	X^2^ = 0.713, d.f. = 1
Minority	68.9% (*n* = 31/45)	65.4% (Adult clinic)	*p* = 0.4017
Hispanic	57.8% (*n* = 26/45)	58.6% (Adult clinic)	
Age (Adult)			
Average Range	41.2 years 18–81 years	42.3 years 18–89 years	Two-tailed *t*-test *p* = 0.711

**Table 2:** Analysis of adult demographics for samples testing positive or negative for HPV.

Demographic Variable	HPV-Negative (*n* = 41/45)	HPV-Positive (*n* = 4/45)	Statistical Analysis: Fisher’s Exact Test
Males (Adult)	43.9% (*n* = 18/41)	50% (*n* = 2/4)	
Females (Adult)	56.1% (*n* = 23/41)	50% (*n* = 2/4)	*p* = 0.8418
Total	91.1% (*n* = 41/45)	8.9% (*n* = 4/45)	
Non-Minority (Adult)	29.3% (*n* = 12/41)	25% (*n* = 1/4)	
Minority (Adult)	70.7% (*n* = 29/41)	75% (*n* = 3/4)	*p* = 0.8573
Total	91.1% (*n* = 41/45)	8.9% (*n* = 4/45)	
Vaccination range	63.4% (*n* = 26/41)	75% (*n* = 3/4)	
Non-vaccination range	36.6% (*n* = 15/41)	25% (*n* = 1/4)	*p* = 0.7869
Average age	43.8 years	41.3 years	

**Table 3:** Analysis of pediatric patient study sample demographics.

Demographic	Study Samples	UNLV-SDM Clinic	Statistical Analysis
Sex (Pediatric)			
Males (Pediatric)	42.9% (*n* = 18/42)	47.2% (Pediatric clinic)	X^2^ = 0.642, d.f. = 1
Females (Pediatric)	57.1% (*n* = 24/42)	52.8% (Pediatric clinic)	*p* = 0.4229
Ethnicity/Race (Pediatric)			
White/Caucasian	16.7% (*n* = 7/42)	24.7% (Pediatric clinic)	X^2^ = 3.413, d.f. = 1
Minority	83.3% (*n* = 35/42)	75.3% (Pediatric clinic)	*p* = 0.0647
Hispanic	54.7% (*n* = 23/42)	52.1% (Pediatric clinic)	
Age (Pediatric)			
Average Range	12.3 years 5–17 years	10.4 years 0–17 years	Two-tailed *t*-test *p* = 0.2331

**Table 4:** Analysis of pediatric demographics for samples testing positive or negative for HPV.

Demographic Variable	HPV-Negative (*n* = 36/42)	HPV-Positive (*n* = 6/42)	Statistical Analysis: Fisher’s Exact Test
Males (Pediatric)	44.4% (*n* = 16/36)	33.3% (*n* = 2/6)	
Females (Pediatric)	55.6% (*n* = 20/36)	66.7% (*n* = 4/6)	*p* = 0.6852
Total	85.7% (*n* = 36/42)	14.3% (*n* = 6/42)	
non-Minority (Pediatric)	19.4% (*n* = 7/36)	33.3% (*n* = 2/6)	
Minority (Pediatric)	80.6% (*n* = 29/36)	66.7% (*n* = 4/6)	*p* = 0.5928
Total	85.7% (*n* = 36/42)	14.3% (*n* = 6/42)	
Vaccination range	66.7% (*n* = 24/36)	100% (*n* = 6/6)	X^2^ = 49.254, d.f. = 1
Non-vaccination range	33.3% (*n* = 12/36)	0% (*n* = 0/6)	*p* = 0.1589
Average age	12.3 years	12.8 years	

## Data Availability

The data presented in this study are available upon request from the corresponding author.
